# Relationship Between Serum Interleukin-6 Levels, Systemic Immune-Inflammation Index, and Other Biomarkers Across Different Rheumatoid Arthritis Severity Levels

**DOI:** 10.7759/cureus.72334

**Published:** 2024-10-24

**Authors:** Shah Zeb, Zahir Khan, Ashraf, Mustafa Javaid, Rumman, Muhammad Arsalan Azmat Swati, Zenab Javaid, Muhammad Luqman

**Affiliations:** 1 Internal Medicine, Bacha Khan Medical College, Mardan, PAK; 2 Orthopaedic Surgery, Medical Teaching Institution Mardan Medical Complex, Bacha Khan Medical College, Mardan, PAK; 3 Research and Development, Pro-Gene Diagnostics and Research Laboratory, Mardan, PAK; 4 Internal Medicine, Oxford University Hospitals NHS Foundation Trust, Oxford, GBR; 5 Active Drug Safety Monitoring and Management (aDSM), Global Fund, Combined Management Unit (CMU), Mardan, PAK; 6 Pulmonology, Medical Teaching Institution Mardan Medical Complex, Mardan, PAK; 7 Orthopaedics and Trauma, Medical Teaching Institution Mardan Medical Complex, Mardan, PAK; 8 General Medicine, Peshawar General Hospital, Peshawar, PAK; 9 Pharmacology and Therapeutics, Peshawar Medical and Dental College, Peshawar, PAK

**Keywords:** bone biomarkers, interleukin-6 (il-6), rheumatoid arthritis, severity of disease, systemic immune-inflammation index (sii)

## Abstract

Background

Rheumatoid arthritis (RA) is a chronic autoimmune disorder characterized by joint inflammation, pain, and progressive disability. Identifying biomarkers that accurately reflect disease severity is crucial for effective management. Interleukin-6 (IL-6) is a pro-inflammatory cytokine involved in the pathogenesis of RA, and the systemic immune-inflammation index (SII) is emerging as a useful marker of systemic inflammation. This study aims to explore the relationship between serum IL-6 levels, SII, and various biomarkers to better predict disease severity in RA patients.

Objective

To determine the relationship between serum IL-6 levels and the SII, along with various biomarkers, across different severity levels for predicting the severity of RA in patients.

Methods

This cross-sectional, observational study was conducted at the Mardan Medical Complex from January 2024 to August 2024, involving 67 RA patients. Clinical assessments included demographic data, disease activity (DAS28), pain (VAS), joint damage (Larsen score), and functional status (HAQ-DI). Serum IL-6 levels, along with other biomarkers such as C-reactive protein (CRP), erythrocyte sedimentation rate (ESR), and the SII, were measured through fasting blood samples. Statistical analyses, including density plots, scatter plots, boxplots with ANOVA, and random forest models, were performed to explore associations between IL-6 and all other variables. Significance was set at p < 0.05.

Results

The study included 67 RA patients (mean age: 41.79 ± 10.51 years, 53.73% male). Elevated IL-6 levels (mean: 80.28 ± 35.27 pg/mL) were strongly associated with disease severity. Patients with DAS28 > 5.5 had IL-6 levels over 100 pg/mL, while those in remission had around 40 pg/mL. IL-6 levels correlated with joint damage (100 pg/mL in severe cases) and pain (over 120 pg/mL for severe pain). Patients with metabolic and cardiovascular comorbidities had the highest IL-6 levels, particularly with diabetes and hypertension (98.6 pg/mL) or cardiovascular disease (119.3 pg/mL). IL-6 correlated strongly with CRP (r = 0.65), ESR (r = 0.51), and SII (r = 0.62). Regression confirmed IL-6 as an independent predictor of severity (p < 0.001), with comorbidities being key predictors.

Conclusion

Elevated IL-6 and SII levels serve as critical markers for predicting the severity of RA. Addressing these markers may lead to more targeted and effective therapeutic strategies for managing disease progression.

## Introduction

Rheumatoid arthritis (RA) is a chronic autoimmune disease characterized by synovial joint inflammation, leading to joint damage, disability, and reduced quality of life [1]. Affecting approximately 0.5%-1% of the global population, RA can occur across all ages and ethnicities, though it is more frequently diagnosed in women [2]. Beyond joint involvement, RA is also linked to systemic inflammation that can affect various organs, including the cardiovascular system, leading to increased morbidity and mortality [3]. Timely diagnosis and effective treatment are crucial in preventing long-term complications and improving patient outcomes.

Interleukin-6 (IL-6), a pro-inflammatory cytokine, plays a key role in the development of RA [4]. It promotes inflammation, autoimmunity, and bone resorption, making it an important biomarker in tracking disease progression [5,6]. Elevated IL-6 levels have consistently been associated with increased disease activity in RA [7], highlighting its central role in the immune-inflammatory response. The Systemic Immune Inflammation Index (SII), which integrates neutrophil, lymphocyte, and platelet counts, has also emerged as a valuable tool for assessing systemic inflammation in RA [8]. Given the critical role of inflammation in RA, the combined use of IL-6 and SII presents a promising approach for predicting disease severity and guiding treatment decisions.

In addition to IL-6 and SII, biomarkers such as C-reactive protein (CRP) and erythrocyte sedimentation rate (ESR) are routinely employed to assess RA disease activity [9,10]. However, these markers have limitations, and there is a need for more reliable biomarkers that can better predict disease severity and patient outcomes. As personalized medicine advances, understanding the interactions between various biomarkers and immune-inflammatory indices is essential for developing tailored treatment strategies for RA patients [11,12].

Despite the established role of IL-6 in RA [13], its correlation with systemic immune inflammation and disease severity is not fully understood. While previous studies have generally focused on individual biomarkers or indices [14,15], comprehensive research on the relationship between serum IL-6 levels and SII across different RA severity levels is lacking. Moreover, the predictive value of combining IL-6 with other inflammatory biomarkers remains inadequately explored.

This gap in knowledge poses challenges for clinicians in predicting RA progression and determining optimal treatment strategies. Current diagnostic approaches rely heavily on clinical assessments and generalized biomarkers [16], which may not fully capture RA's complex inflammatory processes. Therefore, investigating the combined use of IL-6, SII, and other biomarkers as a predictive model for RA severity could provide a deeper understanding of the disease's mechanisms.

Previous research has underscored IL-6’s significant role in RA’s inflammatory cascade. Elevated IL-6 levels are linked to increased joint damage, higher disease activity, and poorer functional outcomes in RA patients [17]. IL-6 inhibitors, such as tocilizumab, have demonstrated effectiveness in reducing inflammation and improving symptoms, further emphasizing IL-6's role in RA pathophysiology [18]. However, while IL-6’s relationship with RA severity is well-documented, its association with other inflammatory indices, such as the SII, remains underexplored.

The SII has gained attention as a marker of systemic inflammation in conditions such as cancer and cardiovascular diseases [19]. In RA, the SII provides a more comprehensive view of the immune response by integrating neutrophil, lymphocyte, and platelet counts [20]. Although some studies have investigated SII's prognostic value in various inflammatory diseases, research on its application in RA is limited.

Other biomarkers, such as CRP and ESR, have long been used to assess RA activity. While these markers offer valuable insights into inflammation, they are not specific to RA and can be influenced by other factors, such as infections or comorbidities [21]. Recent findings suggest that combining multiple biomarkers could enhance diagnostic accuracy and predictive capability in RA [22], but further research is needed to validate this approach.

The primary gap in current research is the limited exploration of the combined use of IL-6, SII, and other biomarkers in predicting RA severity. While individual biomarkers have been extensively studied, a more integrated approach considering the interaction between different immune-inflammatory factors is necessary. Additionally, the potential to use these biomarkers for patient stratification based on disease severity and to guide treatment decisions remains under investigation. Addressing this gap is essential for improving RA management and patient outcomes.

This study seeks to address the question: "How do serum IL-6 levels and the SII, alongside other inflammatory biomarkers, correlate with rheumatoid arthritis severity?" It is hypothesized that elevated IL-6 and SII values will correlate with higher RA severity and that combining these biomarkers will yield a more precise model for predicting disease progression.

This study holds the potential to significantly advance the field of rheumatology by providing new insights into the relationships between IL-6, SII, and RA severity. By evaluating the combined utility of these biomarkers, the study aims to enhance the prediction of RA severity, promote early diagnosis, and support personalized treatment approaches. Additionally, the findings could offer valuable clinical guidance for optimizing RA treatment strategies and improving long-term patient outcomes. The study aims to fill a critical gap in the literature, contributing to a more comprehensive understanding of RA’s inflammatory mechanisms and advancing biomarker-driven diagnostic and therapeutic practices.

## Materials and methods

Study design and setting

This cross-sectional, observational study was conducted at the Mardan Medical Complex Department of Orthopedic and Internal Medicine. The recruitment of participants and data collection took place between January 2024 and August 2024. The study used a convenience sampling technique.

Participant selection

Patients who were diagnosed with RA, based on the 2010 American College of Rheumatology (ACR) and European League Against Rheumatism (EULAR) classification criteria for RA [23], were enrolled in the study. Inclusion and exclusion criteria were established to ensure the recruitment of patients representative of the RA population and to minimize confounding factors.

Inclusion criteria

Patients in this study were aged 18 years or older and had a confirmed diagnosis of RA for at least six months prior to enrollment. Additionally, all individuals provided informed consent, indicating their willingness to participate in the study.

Exclusion criteria

Patients with other inflammatory or autoimmune diseases, such as systemic lupus erythematosus or ankylosing spondylitis, which could influence biomarker levels, as well as individuals who were currently using or had recently (<3 months) used biological therapies targeting IL-6, such as tocilizumab, or other biological disease-modifying antirheumatic drugs (DMARDs), were excluded. Patients with a history of malignancy or any major concurrent illness, including active infections or renal failure, were also not eligible. Pregnant or lactating women, as well as patients unable or unwilling to provide informed consent, were excluded from participation.

Sample size calculation

The sample size for this study was estimated using G*Power 3.1 (The G*Power Team, Germany). To determine the appropriate sample size, a moderate effect size (r = 0.30) was selected based on previous literature that examined the correlation between serum IL-6 levels and RA disease activity indicators. With a significance level (α) of 0.05 and a statistical power of 0.80, G*Power calculated that a minimum of 64 participants would be necessary to detect statistically significant correlations. To account for potential data loss or non-responses, the sample size was slightly increased to 67 participants. This approach ensured the study had sufficient power to detect meaningful associations between IL-6 and clinical outcomes in RA patients, maintaining statistical rigor and reliability in the findings. However, no patient data were lost during the study.

Clinical assessments

All patients underwent comprehensive clinical evaluation at the time of enrollment. This evaluation included demographic data, clinical characteristics, and standardized measures of RA disease activity, joint damage, and functional status. The assessments were performed by a trained doctor to ensure accuracy and consistency.

Demographic and clinical data

Patient data included several key variables. Age, gender, and BMI were calculated by dividing weight in kilograms by height in meters squared and categorized as normal, overweight, or obese, according to World Health Organization (WHO) guidelines [24]. The duration of RA was assessed based on the number of years since the patient's diagnosis, as reported by the patient and verified through medical records. Morning stiffness, a common symptom of RA, was reported by patients in terms of duration in minutes. Additionally, a 28-joint count was performed by a rheumatologist to record the total number of swollen and tender joints, providing a detailed assessment of joint involvement.

Disease severity categorization

Disease activity in RA was assessed using several standardized measures. The Disease Activity Score in 28 Joints (DAS28), a composite index incorporating swollen and tender joint counts, erythrocyte sedimentation rate (ESR), and patient-reported global health status, was used to categorize disease activity into remission, low, moderate, or high levels [25]. The Physician’s Global Assessment of Disease Severity was rated by the rheumatologist on a visual analog scale (VAS) from 0 (no disease activity) to 4 (maximum disease activity) [26]. For pain evaluation, patients rated their pain over the past week using a VAS, where 0 represented no pain and 10 represented the worst possible pain [27]. Functional disability was measured using the Health Assessment Questionnaire Disability Index (HAQ-DI), which assessed patients' difficulty in performing daily activities, with scores ranging from 0 (no disability) to 3 (severe disability) [28]. To assess joint damage, radiographs of the hands, wrists, and feet were taken, and the Larsen severity score was applied, grading joint destruction from 0 (no damage) to 5 (complete joint destruction).

Laboratory measurements

All laboratory measurements were conducted on fasting blood samples obtained during the clinical evaluation of each patient. Biomarker analyses included both routine clinical markers and advanced inflammatory markers relevant to RA. Hemoglobin, white blood cell (WBC) count, and platelet count were measured using automated hematology analyzers using Horiba Yumizen H550, while creatinine and urea were assessed to monitor renal function using roach cobas c111. Inflammatory biomarkers, such as CRP and ESR, were measured using ElectroChemiLuminescence assays roach cobas e411 and the Westergren method, respectively, with elevated CRP levels (>10 mg/L) indicating active inflammation. IL-6 was quantified using enzyme-linked immunosorbent assays (ELISA) using ELK biotechnology ELISA kits. Additionally, rheumatoid factor (RF) was measured via latex-enhanced immunonephelometry Genrui PA50, with levels above 20 IU/mL considered positive. The SII was calculated using the formula SII=Neutrophil count×Platelet countLymphocyte count\text{SII} = \frac{\text{Neutrophil count} \times \text{Platelet count}}{\text{Lymphocyte count}}SII=Lymphocyte countNeutrophil count×Platelet count​, serving as a validated marker for systemic inflammation in RA [8].

Statistical analysis

Data analysis was performed using R version 4.4.1 (R Development Core Team, Vienna, Austria), with a p-value < 0.05 considered statistically significant. Descriptive statistics summarized continuous variables were summarized as mean ± standard deviation, and categorical variables were expressed as frequencies and percentages. Heatmaps were used to visualize the relationships between IL-6 levels, the SII, and clinical outcomes including DAS28, Physician’s Global Assessment, HAQ-DI, VAS pain severity, and Larsen severity, providing a comparative, visual interpretation of inflammation and disease severity. Multiple linear regression models, adjusted for age, gender, BMI, disease duration, and comorbidities, assessed the independent association between IL-6 levels and disease severity outcomes such as DAS28, HAQ-DI, and Larsen scores, with diagnostic plots such as fitted vs. residuals and scale-location plots evaluating model assumptions. Boxplots with ANOVA tested differences in IL-6 levels across disease severity groups, with post-hoc Tukey’s tests applied for pairwise comparisons. Density plots were used to show IL-6 level distributions across RA duration, swollen joint count, tender joint count, and morning stiffness duration categories. The impact of comorbidities on IL-6 was analyzed using random forest models and radar plots, identifying significant predictors of elevated IL-6 levels. Diagnostic plots, including residual plots, Q-Q plots, and leverage plots, were used to ensure model validity, checking for normality of residuals, homoscedasticity, and influential data points.

Ethical considerations

The study protocol was approved by the Institutional Review Board (IRB) No: 634/BKMC of the hospital where the study was conducted. All participants provided written informed consent prior to enrollment. Confidentiality of patient data was ensured by assigning unique identification numbers to each participant. Data were stored securely and were accessible only to the study investigators.

## Results

The study included 67 RA patients with a mean age of 41.79 ± 10.51 years, slightly more males (53.73%) than females (46.27%), and a mean body mass index (BMI) of 26.43 ± 3.19, placing most in the overweight category. Patients had been diagnosed with RA for an average of 6.91 ± 2.39 years, with a mean swollen joint count of 7.42 ± 2.41 and a tender joint count of 9.90 ± 3.21, indicating significant joint involvement (Table 1).

**Table 1:** Demographic and Clinical Characteristics of Rheumatoid Arthritis Patients, Including Biomarkers and Comorbidities Data are presented as mean and standard deviation or frequency and percentages.

Characteristics	All patients
Total no of patients	67 (100%)
Age	41.79±10.51
Gender	
Male	36 (53.73%)
Female	31 (46.27%)
BMI	26.43±3.19
Duration of RA diagnosis in years	6.91±2.39
Swollen joint count	7.42±2.41
Tender joint count	9.90±3.21
Presence of morning stiffness (duration in minutes)	74.28±24.14
Comorbidities	
Cardiovascular disease	01 (1.49%)
Chronic pulmonary obstructive diseases	01 (1.49%)
Diabetes mellitus	10 (14.93%)
Diabetes mellitus + Chronic pulmonary obstructive diseases	01 (1.49%)
Diabetes mellitus + Hypertension	03 (4.48%)
Diabetes mellitus + Hypertension +Cardiovascular Disease	02 (2.99%)
Hypertension + Cardiovascular Disease	01 (1.49%)
Biomarkers	
Hemoglobin levels (g/dL)	10.56±1.36
White blood cell count (WBC) (x10^9^/L)	12.38±4.02
Neutrophils (u/L)	8443.44±3022.51
Lymphocytes (u/L)	2901.98±726.23
Monocytes (u/L)	686.46±241.32
Basophils (u/L)	95.74±40.47
Eosinophils (u/L)	252.51±29.87
Platelet count (x10^9^/L)	345.31±69.61
Creatinine levels (mg/dL)	1.19±0.08
Urea levels (mg/dL)	29.90±3.21
Rheumatoid factor (RF) levels (IU/mL)	50.28±15.26
Erythrocyte Sedimentation Rate (ESR) (mm/hr)	48.57±20.47
C-Reactive Protein (CRP) (mg/L)	42.82±23.48
Systemic Immune-Inflammatory Index (SII)	946.61±248.13
IL-6 levels (Serum) (pg/mL)	80.28±35.27

The Disease Activity Score (DAS28) showed that nearly half of the patients (48.76%) had high disease activity, with 34.33% presenting moderate disease activity, while remission was observed in only 4.48% of patients. Similarly, the Physician’s Global Assessment of Disease Severity aligned closely, with 46.27% of patients categorized as having moderate disease activity and 22.39% classified as having high disease activity, while remission or very low disease activity was rare (4.48%). Functional disability, as assessed by the Health Assessment Questionnaire Disability Index (HAQ-DI), revealed that 38.81% of patients had mild disability, while 35.82% exhibited moderate disability, and a smaller percentage (16.42%) had severe disability, highlighting the varying impact of RA on daily functioning. In terms of joint pain severity, as measured by the VAS, the majority of patients reported moderate (43.29%) or severe (34.33%) pain, with 17.91% experiencing very severe pain, and none of the patients were pain-free. Radiographic assessments using the Larsen severity score indicated that more than half of the patients (52.24%) had very severe joint damage, while 22.39% had moderate damage and 20.90% had severe joint damage, further underscoring the advanced nature of the disease in this cohort (Table 2).

**Table 2:** Clinical and Functional Assessment of Disease Activity, Disability Levels, Pain Severity, and Radiographic Severity in RA Patients Data are presented as frequency and percentages.

Characteristics	All patients
All patients	67 (100%)
Disease activity severity (DAS28)	
Remission	03 (4.48%)
Low Disease Activity	09 (13.43%)
Moderate Disease Activity	23 (34.33%)
High Disease Activity	32 (48.76%)
Physician's global assessment of disease severity	
Remission or very low disease activity	03 (4.48%)
Mild disease activity	18 (26.87%)
Moderate disease activity	31 (46.27%)
High disease activity	15 (22.39%)
Functional disability ( HAQ-DI - Health Assessment Questionnaire Disability Index)	
No Disability	06 (8.96%)
Mild Disability	26 (38.81%)
Moderate Disability	24 (35.82%)
Severe Disability	11 (16.42%)
Joint pain severity (VAS score)	
No pain	00 (0.00%)
Mild pain	03 (4.48%)
Moderate pain	29 (43.29%)
Severe pain	23 (34.33%)
Very severe pain	12 (17.91%)
Larsen Severity	
Mild	03 (4.48%)
Moderate	15 (22.39%)
Severe	14 (20.90%)
Very severe	35 (52.24%)

**Figure 1:**
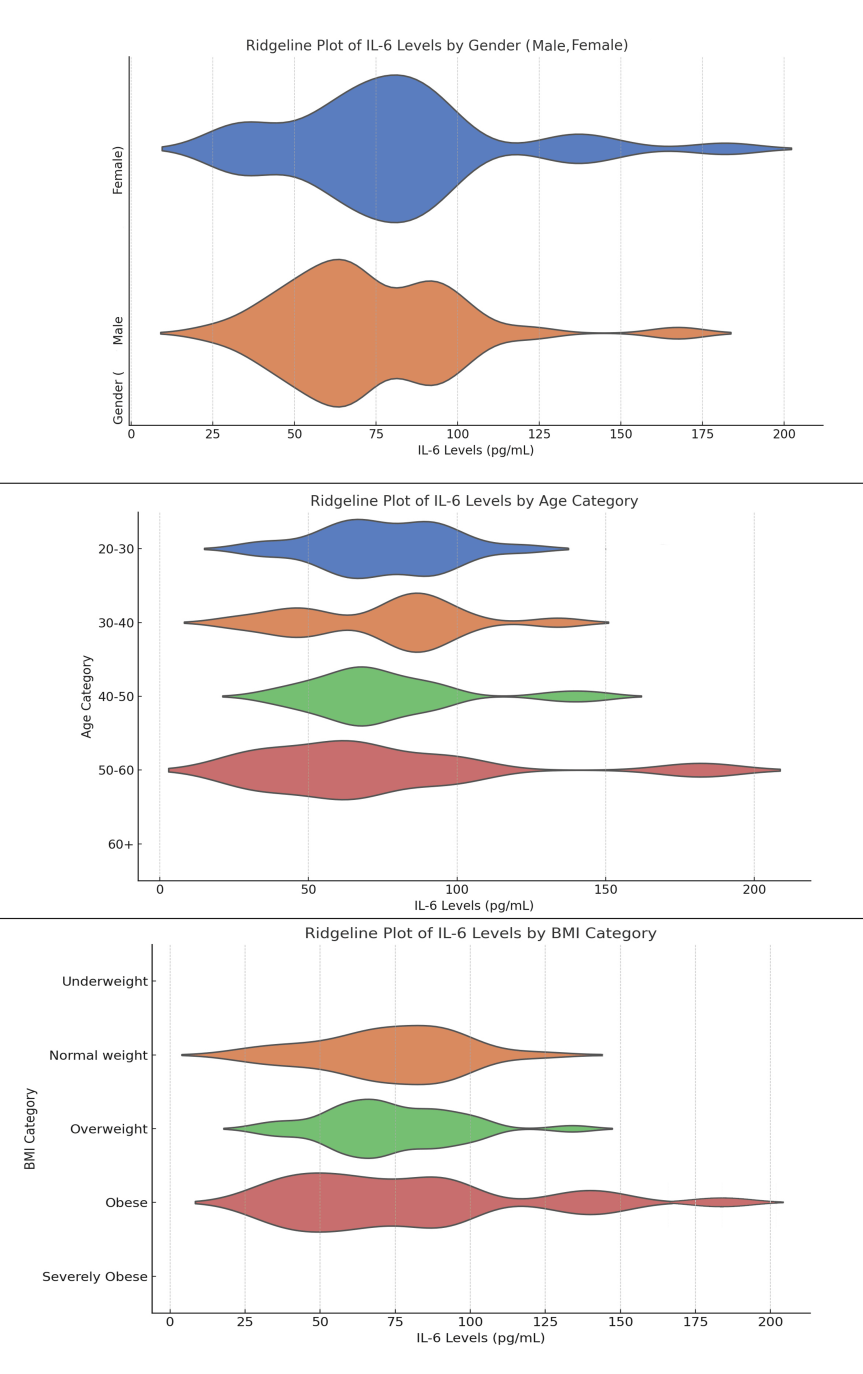
Distribution of Serum IL-6 Levels Across Gender, Age, and BMI Categories in Rheumatoid Arthritis Patients: A Ridgeline Plot Analysis

The ridgeline plots of IL-6 levels across gender, age, and BMI categories reveal notable variations in inflammatory responses among RA patients. Females exhibit a broader range of IL-6 levels, with peaks between 50 and 100 pg/mL and cases extending to 150-200 pg/mL, while males show a narrower distribution centered around 50-100 pg/mL. Age-wise, younger patients (20-30 years) display a wider range of IL-6 levels, with older patients (50-60 and 60+) showing broader distributions up to 200 pg/mL, suggesting increased inflammation with age. BMI analysis highlights that patients in overweight and obese categories have elevated IL-6 levels, with severely obese individuals showing levels up to 200 pg/mL, indicating a potential link between higher BMI and greater inflammatory activity (Figure 1).

**Figure 2:**
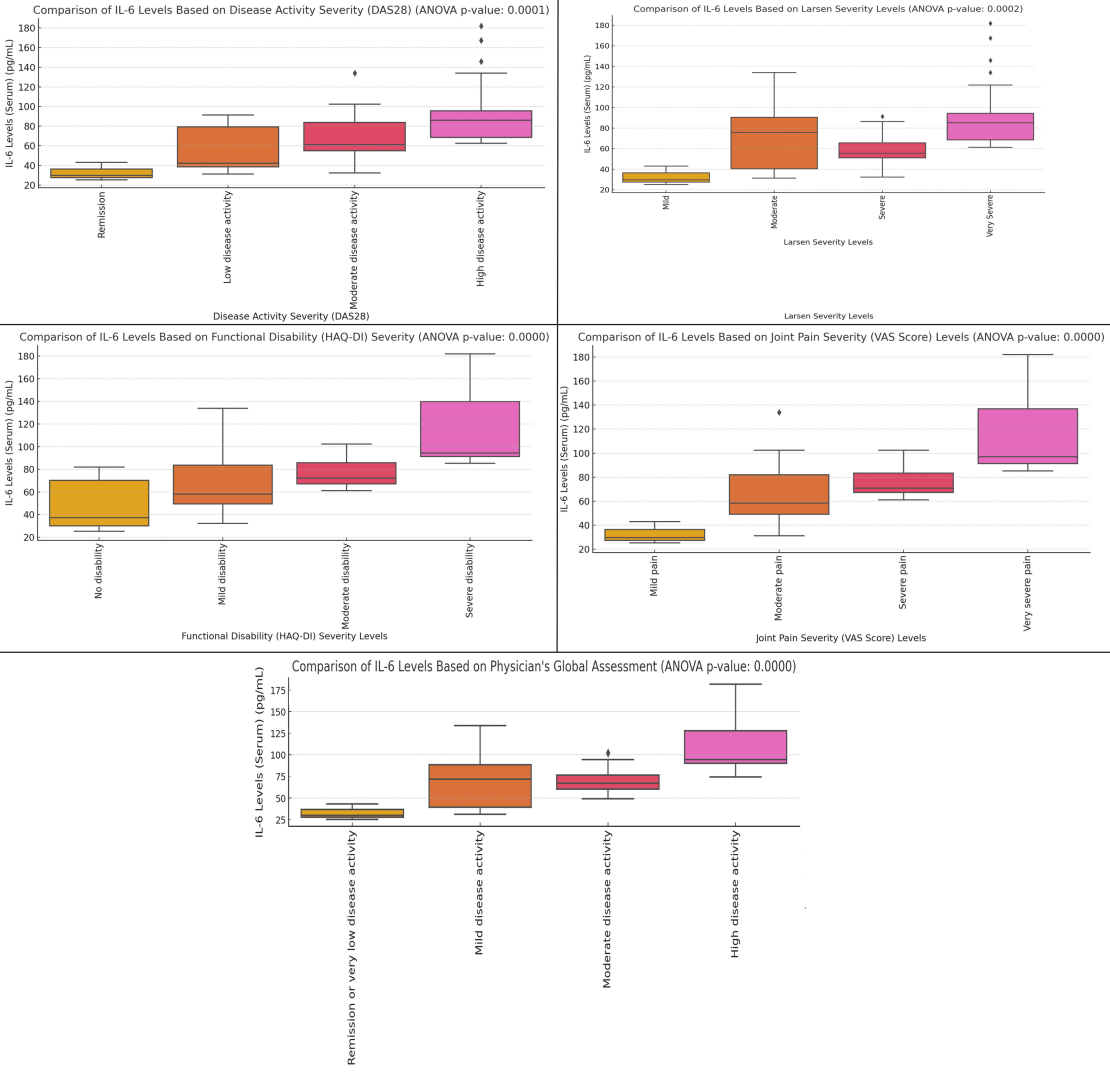
Comparative Analysis of IL-6 Levels Across Disease Activity, Joint Damage, Functional Disability, Pain Severity, and Physician's Assessment in Rheumatoid Arthritis Patients A one-way ANOVA statistical test was conducted to detect differences between the groups, with a p-value of less than 0.05 deemed statistically significant.

The figure illustrates a clear relationship between IL-6 levels and various clinical measures in RA patients, with all comparisons showing significant associations (ANOVA p-values < 0.0001). In terms of disease activity (DAS28), IL-6 levels rise progressively with increasing disease severity: patients in remission have median IL-6 levels around 40 pg/mL, those with low disease activity show levels near 50 pg/mL, moderate disease activity corresponds to approximately 75 pg/mL, and high disease activity exceeds 100 pg/mL. For the Larsen severity of joint damage, patients with mild joint damage exhibit IL-6 levels around 40 pg/mL, moderate damage shows levels close to 75 pg/mL, and very severe joint damage is associated with IL-6 levels around 100 pg/mL. The functional disability (HAQ-DI) plot shows a similar pattern, with no disability corresponding to IL-6 levels of 40 pg/mL, mild disability to 60 pg/mL, moderate disability to 75 pg/mL, and severe disability peaking at nearly 100 pg/mL. In Joint Pain Severity (VAS), IL-6 levels increase steadily from around 40 pg/mL for mild pain to 75 pg/mL for moderate pain, 100 pg/mL for severe pain, and exceeding 120 pg/mL for very severe pain. Finally, in the Physician’s Global Assessment, patients in remission or with very low disease activity show IL-6 levels around 40 pg/mL, mild disease activity corresponding to approximately 60 pg/mL, moderate disease activity to 75 pg/mL, and high disease activity to levels exceeding 100 pg/mL (Figure 2).

**Figure 3:**
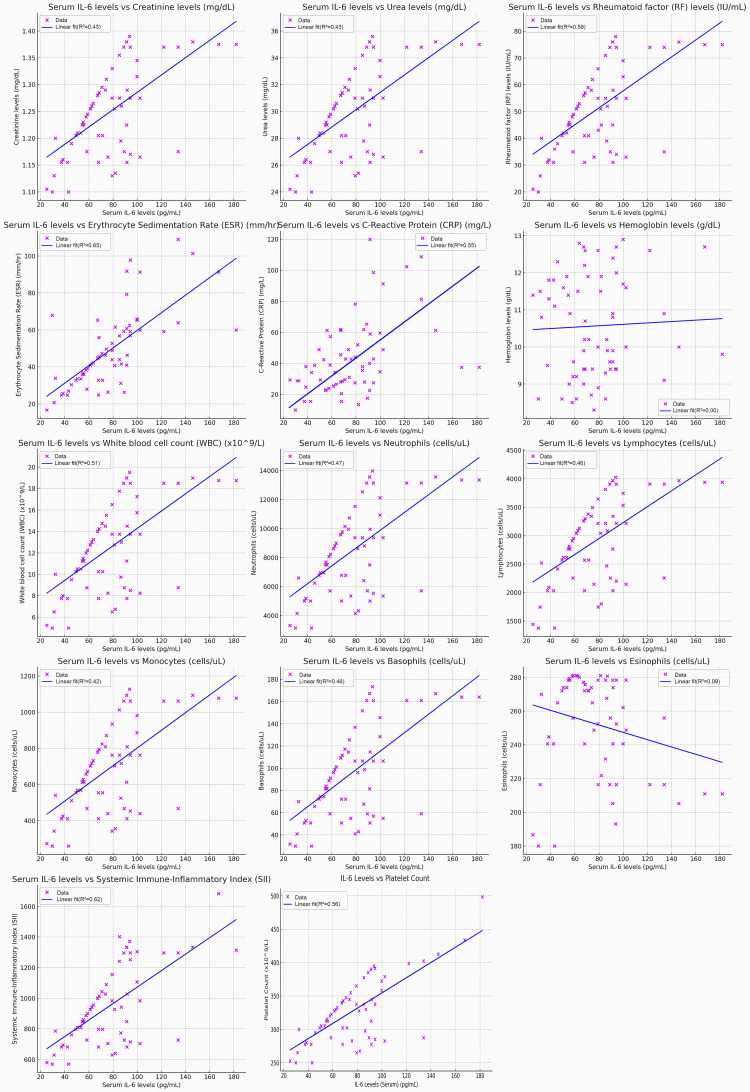
Relationship of Serum IL-6 Levels with all Biomarkers, and Systemic Inflammatory Markers in Rheumatoid Arthritis Patients

The figure presents scatter plots showing the correlation between serum IL-6 levels and various biomarkers in RA patients. Creatinine levels and urea levels both show moderate correlations with IL-6 levels, each with an R² of 0.43, indicating a link between IL-6 and kidney function. IL-6 has a stronger positive correlation with RF (R² = 0.59), suggesting its significant association with autoimmune activity in RA. The inflammatory markers ESR (R² = 0.65) and CRP (R² = 0.51) demonstrate substantial positive correlations, confirming IL-6’s role as a key indicator of systemic inflammation. The WBC count also correlates positively with IL-6 (R² = 0.51), and a similar correlation is seen with neutrophils (R² = 0.47), emphasizing IL-6’s association with immune cell activation. Lymphocytes (R² = 0.48) and monocytes (R² = 0.42) also show moderate correlations, while basophils (R² = 0.46) have a slightly stronger correlation with IL-6, all of which suggest that IL-6 is related to various immune cell activities. In contrast, hemoglobin levels exhibit no correlation (R² = 0.00), and eosinophils show a weak negative correlation (R² = 0.09), indicating minimal involvement of these markers with IL-6. The SII shows a strong positive correlation with IL-6 (R² = 0.62), highlighting IL-6's role in systemic immune activation. Finally, platelet count also correlates positively with IL-6 (R² = 0.56), indicating IL-6’s contribution to thrombocytosis in the inflammatory process (Figure 3).

**Figure 4:**
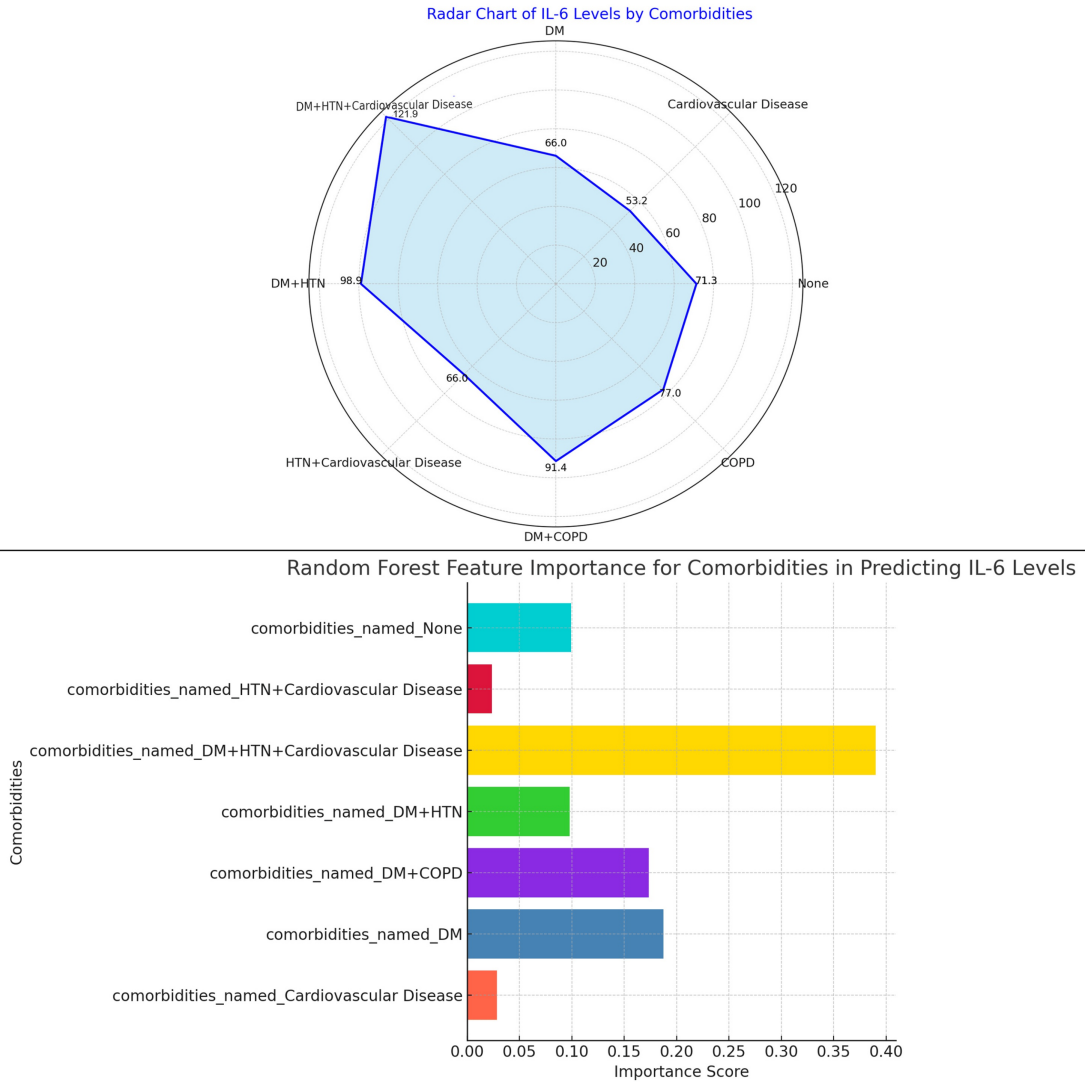
Impact of Comorbidities on IL-6 Levels in Rheumatoid Arthritis: Radar Chart and Feature Importance Analysis Using Random Forest

The figure presents both a radar chart and random forest feature importance analysis to evaluate the impact of comorbidities on IL-6 levels in RA) patients. The radar chart shows that patients with DM + HTN + cardiovascular disease have the highest IL-6 levels (119.3 pg/mL), followed by patients with DM + HTN (98.6 pg/mL) and HTN + cardiovascular disease (91.4 pg/mL). Patients with DM + COPD and cardiovascular disease exhibit moderate IL-6 levels at 71.0 pg/mL and 66.0 pg/mL, respectively. The lowest IL-6 levels are observed in patients with no comorbidities (71.3 pg/mL). The random forest feature importance analysis highlights that DM + HTN + cardiovascular disease is the most significant predictor of elevated IL-6 levels, with the highest importance score, followed by DM + COPD and DM + HTN, while patients with no comorbidities and those with only cardiovascular disease show lower predictive importance (Figure 4).

**Figure 5:**
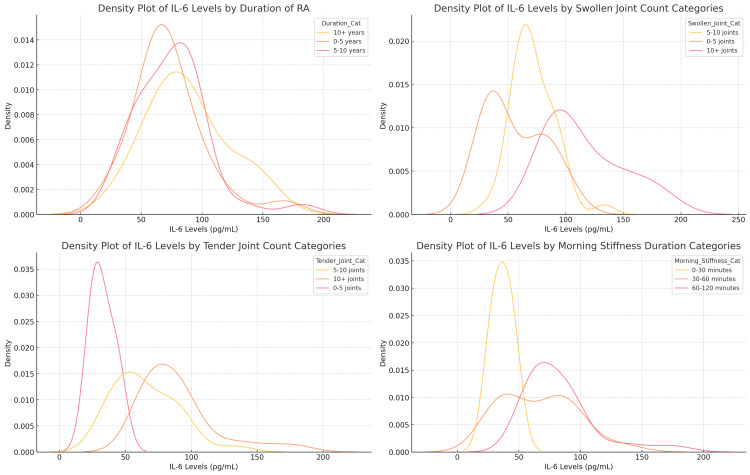
Density Plots of IL-6 Levels by RA Duration, Morning Stiffness, Swollen Joint Count, and Tender Joint Count Categories

The density plots illustrate the distribution of IL-6 levels across various categories of RA duration, joint involvement, and morning stiffness, highlighting the relationship between IL-6 levels and disease severity. Patients with RA for 0-5 years had the highest IL-6 peak at 65-70 pg/mL, while those with RA for 5-10 years peaked at 55-60 pg/mL, and patients with 10+ years of RA showed a peak around 50 pg/mL, but with a wider distribution extending up to 150 pg/mL, indicating increased variability over time. IL-6 levels were higher in patients with more swollen joints, peaking at 100 pg/mL in those with 10+ swollen joints, compared to 70-75 pg/mL in those with 5-10 swollen joints and 50 pg/mL in those with 0-5 swollen joints. Similarly, patients with more tender joints (10+) exhibited a broader IL-6 distribution, peaking at 80-90 pg/mL, while those with fewer tender joints (0-5) had the lowest peak at 35 pg/mL. Finally, IL-6 levels were lowest in patients with 0-30 minutes of morning stiffness (35-40 pg/mL), while patients with 30-60 minutes peaked at 50-60 pg/mL, and those with 60-120 minutes of morning stiffness had the highest IL-6 levels, peaking at 80-90 pg/mL and extending beyond 150 pg/mL (Figure 5).

**Figure 6:**
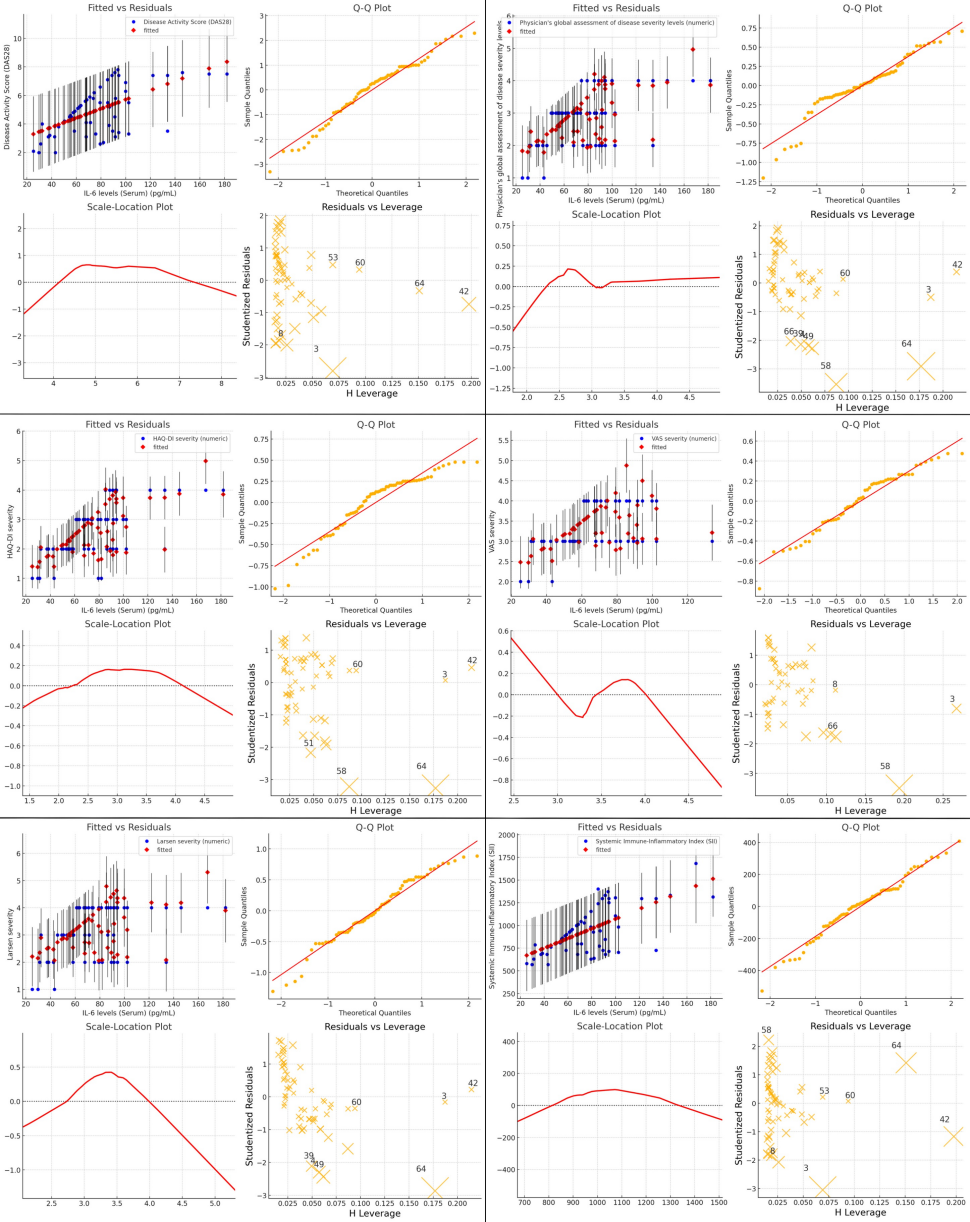
Diagnostic Plots for Regression Analysis of IL-6 Levels Against Disease Severity levels and Systemic Immune-Inflammatory Index (SII)

The diagnostic plots assess the regression fit of IL-6 levels against various clinical outcomes, such as DAS28, Physician’s Global Assessment of Disease Severity, HAQ-DI severity, VAS severity, Larsen severity, and SII. The Fitted vs Residuals plots show an upward trend, indicating a strong correlation between increasing IL-6 levels and higher disease severity, but the spread of residuals grows at higher IL-6 levels, suggesting variability and reduced model precision for extreme IL-6 concentrations. The Q-Q plots confirm that the residuals generally follow a normal distribution, though deviations are observed at the extremes. The scale-location plots reveal that variance increases with IL-6 levels, implying better model fit at lower to moderate IL-6 values. The residual vs leverage plots identifies patients (notably 42, 60, and 64) with high leverage, indicating that these outliers may disproportionately affect the model. IL-6 strongly correlates with DAS28, physician assessments, HAQ-DI, VAS, Larsen severity, and SII, but higher IL-6 levels introduce variability across outcomes. Overall, while IL-6 serves as a robust predictor of RA severity and inflammation, further investigation is needed for patients with very high IL-6 levels to understand additional influencing factors. These findings highlight IL-6's central role in RA management but also point to heterogeneity in patient responses (Figure 6).

**Figure 7:**
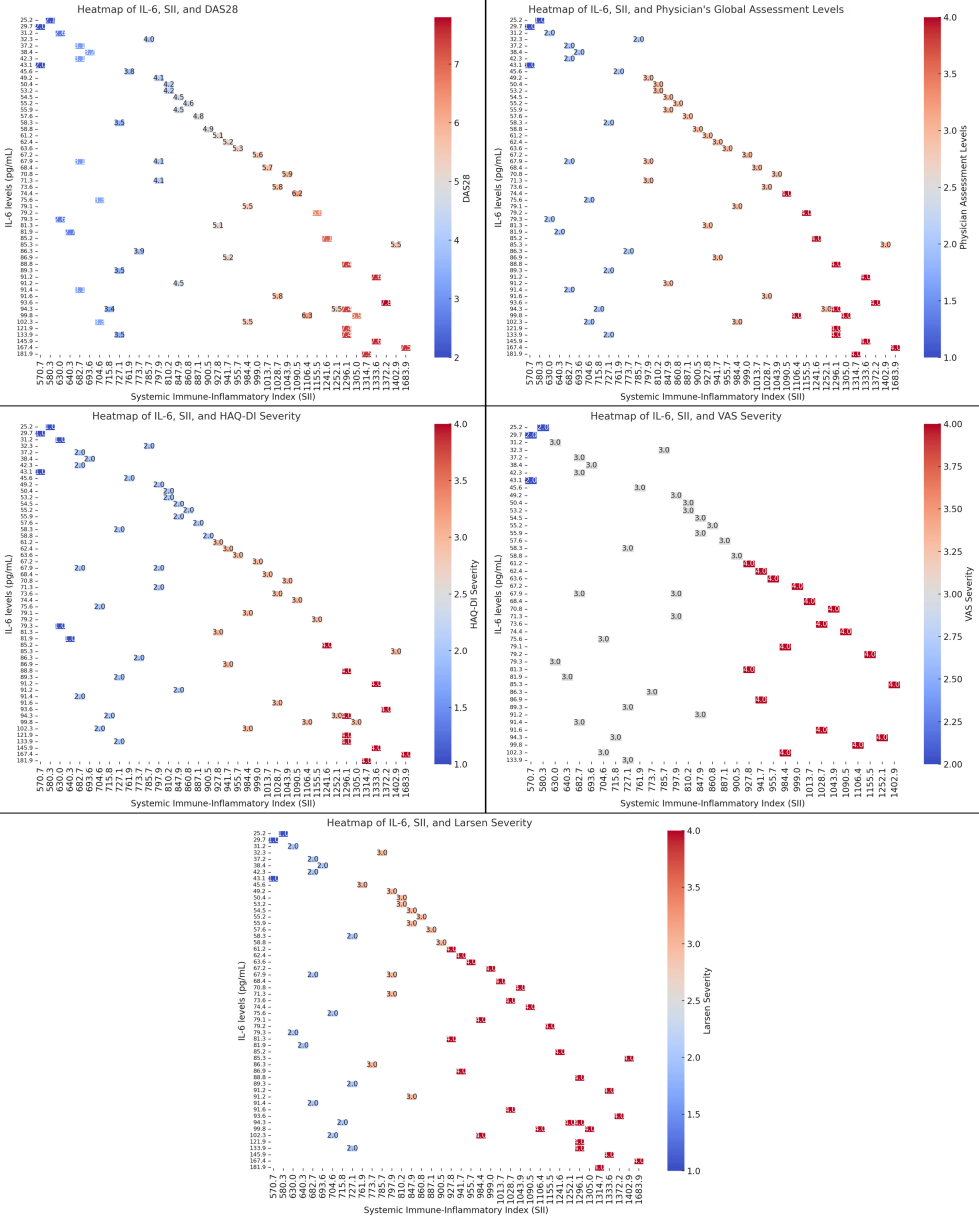
Heatmap Analysis of IL-6 Levels, Systemic Immune-Inflammatory Index (SII), and Clinical Outcomes in Rheumatoid Arthritis Patients

The heatmaps illustrate the relationship between IL-6 levels, the SII, and several clinical outcomes in RA patients, including DAS28, Physician’s Global Assessment of Disease Severity, HAQ-DI, VAS severity, and Larsen severity. As IL-6 levels exceed 80 pg/mL, corresponding DAS28 scores rise above 5.5, indicating severe disease activity, while IL-6 levels between 40-50 pg/mL align with DAS28 scores of 3.5-4.5, reflecting moderate activity. Similarly, IL-6 levels over 80 pg/mL result in the Physician’s Global Assessment of Disease Severity scores of 3.0-3.5, while lower IL-6 levels correspond to scores of 2.0-2.5. For HAQ-DI, IL-6 levels above 80 pg/mL relate to severe disability scores of 3.5-4.0, while lower levels (around 40-50 pg/mL) correspond to milder disability scores of 2.0-2.5. Patients with IL-6 levels above 80 pg/mL also report higher pain scores (VAS 3.5-4.0), while those with 40-50 pg/mL report milder pain (VAS 2.5-3.0). Joint damage, as measured by Larsen scores, is similarly linked to higher IL-6 levels, with scores around 3.0-3.5 for IL-6 levels above 80 pg/mL and 2.0-2.5 for IL-6 levels around 40-50 pg/mL. Across all heatmaps, increased IL-6 and SII levels are consistently associated with worse clinical outcomes, reinforcing the central role of IL-6 as a biomarker for inflammation, disease severity, and progression in RA patients (Figure 7).

## Discussion

RA is a complex autoimmune disorder characterized by chronic inflammation, primarily affecting the synovial joints, leading to pain, swelling, stiffness, and eventual joint destruction. The pathophysiology of RA involves a myriad of inflammatory mediators, among which interleukin-6 (IL-6) plays a pivotal role. In our study involving 67 RA patients, we aimed to elucidate the relationship between serum IL-6 levels and disease severity while also considering the impact of comorbidities and systemic inflammatory indices on disease progression.

The demographic characteristics of our cohort revealed a mean age of 41.79 ± 10.51 years, with a gender distribution of 53.73% males and 46.27% females. This finding contrasts with previous literature that indicates a higher prevalence of RA among females, as noted by Dahamsheh et al. [29]. The discrepancy may stem from variations in population characteristics or sampling strategies. The patients had a mean BMI of 26.43 ± 3.19, indicating a predominance of overweight individuals, which aligns with findings by Giles et al. [30], who reported that higher BMI is associated with worse RA outcomes due to the pro-inflammatory cytokines released by adipose tissue [31].

Clinical assessments indicated significant joint involvement, with an average swollen joint count of 7.42 ± 2.41 and a tender joint count of 9.90 ± 3.21. This level of joint involvement is indicative of high disease activity, corroborated by the average duration of morning stiffness, which lasted 74.28 ± 24.14 minutes. We find that morning stiffness exceeding 60 minutes is a hallmark of RA, reflecting ongoing joint inflammation. The severity of joint involvement underscores the necessity for early and aggressive treatment strategies to mitigate further joint damage [32].

In our biomarker analysis, we observed elevated levels of CRP at 42.82 ± 23.48 mg/L and ESR at 48.57 ± 20.47 mm/hour, both of which are established indicators of systemic inflammation in RA [33]. Notably, serum IL-6 levels were significantly elevated at 80.28 ± 35.27 pg/mL, reinforcing its role as a central mediator in the inflammatory cascade associated with RA [34]. Previous studies have similarly identified IL-6 as a key player in promoting synovial inflammation and joint destruction [35]. The elevated WBC count of 12.38 ± 4.02 x 10^9^/L, particularly the neutrophil count, further reflects the ongoing immune response in our study. A study found that increased neutrophil counts correlate with heightened disease severity and poorer long-term outcomes in RA patients [36].

Our findings indicate a clear relationship between IL-6 levels and RA disease activity. Higher IL-6 levels were associated with more severe DAS28, higher scores in the Physician’s Global Assessment, and greater functional disability as measured by the HAQ-DI. Patients with IL-6 levels exceeding 80 pg/mL consistently exhibited DAS28 scores above 5.5, indicative of severe disease activity. This observation aligns with previous studies, where elevated IL-6 levels have been linked to increased disease activity and adverse clinical outcomes in RA patients [37]. Furthermore, the correlation between IL-6 and radiographic joint damage, assessed via Larsen scores, supports the notion that IL-6 is a significant driver of joint destruction in RA [38].

The SII, calculated from neutrophil, lymphocyte, and platelet counts, demonstrated a strong positive correlation with IL-6 (R² = 0.62), highlighting IL-6's role in systemic inflammation [39]. Our findings suggest that SII may serve as a complementary marker to IL-6 in evaluating the inflammatory burden in RA patients. The radar chart and random forest analysis revealed that RA patients with comorbid conditions, particularly diabetes mellitus (DM), hypertension (HTN), and cardiovascular disease, exhibited significantly elevated IL-6 levels. The highest IL-6 levels were recorded in patients with DM, HTN, and cardiovascular disease (119.3 pg/mL), consistent with existing research indicating that comorbidities exacerbate inflammation in RA patients [40]. Notably, cardiovascular disease remains a leading cause of mortality in RA patients, with IL-6 implicated in the development of atherosclerosis [41].

Pain severity, as measured by the VAS, showed a clear correlation with IL-6 levels. Patients experiencing moderate to severe pain had elevated IL-6 levels, often exceeding 100 pg/mL. This finding aligns with studies demonstrating IL-6's role in mediating pain and hyperalgesia in inflammatory conditions, including RA [42]. IL-6 acts on peripheral nociceptors and contributes to central sensitization, amplifying pain perception in patients with active inflammation [43]. Targeting IL-6 through biological therapies could potentially reduce inflammation and alleviate pain in RA patients.

Functional disability, assessed via HAQ-DI, was strongly associated with IL-6 levels. Patients with higher IL-6 levels consistently exhibited worse HAQ-DI scores, indicating more severe limitations in daily functioning. This finding is corroborated by existing literature highlighting IL-6's role in mediating joint damage and functional impairment in RA [44]. Therapeutic interventions aimed at reducing IL-6 levels have been shown to improve functional outcomes in RA patients, reinforcing the importance of IL-6 as a therapeutic target.

Comparative analysis with existing literature reveals that our findings are consistent with numerous studies emphasizing IL-6's central role in RA pathophysiology. Saito et al. [45] demonstrated that blocking IL-6 signaling with tocilizumab, a monoclonal antibody targeting the IL-6 receptor, significantly reduced disease activity and improved clinical outcomes in RA patients. Similarly, Malattia et al. [46] reported that IL-6 blockade not only reduced inflammation but also slowed the radiographic progression of joint damage. Our study also underscores the importance of managing comorbidities in RA, particularly metabolic and cardiovascular conditions, which contribute to increased mortality [47].

The strong correlation between IL-6 and functional disability, pain severity, and systemic inflammation emphasizes IL-6's significance as a therapeutic target. Current guidelines recommend biologic therapies, such as tocilizumab, for RA patients with moderate to severe disease activity who have failed conventional treatments [48]. Our findings support the use of IL-6 blockade in this population, particularly for patients with high IL-6 levels and multiple comorbidities.

Limitations

The study has several limitations that should be acknowledged. First, the relatively small sample size of 67 RA patients limits the generalizability of the findings to larger populations or different demographic groups. Additionally, the cross-sectional design of the study prevents the establishment of causal relationships between IL-6 levels and disease severity, which would require longitudinal studies for confirmation. Comorbidities such as diabetes and hypertension, although accounted for, may have influenced IL-6 levels, making it difficult to isolate the effects of RA on systemic inflammation. The single-center nature of the study may also limit the applicability of the findings to different healthcare settings or regions. Furthermore, the study focused on a limited range of biomarkers, excluding other important inflammatory markers such as TNF-α and IL-1, which might have provided a more comprehensive understanding of the inflammatory processes in RA. Future studies should consider employing larger, multi-center cohorts to improve generalizability and include longitudinal designs to establish causal links between biomarkers and disease outcomes. Additionally, expanding the biomarker panel and investigating the influence of comorbidities on inflammatory markers in RA could provide deeper insights into disease mechanisms and improve personalized treatment strategies.

## Conclusions

In conclusion, our study demonstrates that elevated IL-6 levels are closely associated with disease severity, joint damage, functional disability, and pain in RA patients. IL-6 plays a central role in the inflammatory cascade in RA, and its levels correlate strongly with other inflammatory biomarkers and clinical outcomes. Patients with multiple comorbidities, particularly those with metabolic and cardiovascular conditions, are at higher risk of elevated IL-6 levels and worse disease outcomes. Targeting IL-6 through biological therapies may help control disease activity, reduce pain, and improve functional outcomes in RA patients. Our findings underscore the importance of comprehensive management strategies that address both RA and its associated comorbidities to optimize patient outcomes.
